# How does culinary medicine training impact the diet-related knowledge, skills and attitudes of undergraduate medical students in Germany?—A systematic review

**DOI:** 10.1186/s12909-026-09580-2

**Published:** 2026-06-03

**Authors:** Beate Stock-Schröer, Friedrich Edelhäuser, Christian Scheffer, Lea Schweigmann, Angelika Homberg

**Affiliations:** 1 Health Department, Interprofessional Graduate School Integrative Medicine and Health Sciences, Witten/Herdecke University, Witten, Germany; 2 Health Department, Integrated Curriculum Anthroposophical Medicine, Witten/Herdecke University, Witten, Germany; 3 Health Department, Witten/Herdecke University, Witten, Germany; 4 Department of Medical Education Research, Division for Study and Teaching Development, Medical Faculty Mannheim, Heidelberg University, Mannheim, Germany

**Keywords:** Medical education, Interprofessional, Teaching evaluation, Curriculum, Nutrition, Practical Skills

## Abstract

**Background:**

Although nutrition competencies are essential for physicians, nutrition education is underrepresented in German medical schools. Culinary medicine (CM) training and teaching kitchens (TK) are emerging as innovative educational interventions to address this issue. This systematic review summarises the existing literature on teaching projects at German medical schools. Secondly, it provides an overview of the impact of various CM training approaches on the diet-related knowledge, skills and attitudes of German undergraduate medical students, and how participants evaluated the courses overall. The quality of the included studies is also assessed.

**Methods:**

From October to December 2023, we conducted a systematic search of several databases, including PubMed, Web of Science, Scopus, and Embase, for studies involving CM interventions and TK among German undergraduate medical students. A follow-up search was conducted from 25 July to 30 August 2025. We included studies that measured the impact of an interactive teaching on diet-related competencies. Two reviewers independently selected studies, extracted data and assessed study quality using MMERSQI. The various implementation forms and teaching formats were compiled in a descriptive manner. The results of the individual studies were weighted and presented in tables.

**Results:**

Nine studies of six study sites met the inclusion criteria. All interventions were elective modules integrated into clinical education. All studies reported improvements in diet-related knowledge and/or more positive attitudes toward nutrition counselling in clinical practice. No adverse effects were reported. The range of courses on offer was rated as 'satisfactory' or 'very satisfactory' by students at all study sites. Study quality was moderate (range 42–70.5 out of 100; median 60).

**Conclusion:**

Training in CM can effectively enhance the diet-related knowledge, skills and attitudes of German undergraduate medical students. Integrating a programme into the curriculum could provide future doctors with the necessary tools to combat diet-related diseases effectively.

**Trial registration:**

PROSPERO registration number: CRD420251109133.

**Supplementary Information:**

The online version contains supplementary material available at 10.1186/s12909-026-09580-2.

## Background

### Diet-related diseases and prevention

Diet-related diseases are a significant challenge to healthcare systems worldwide. They cause substantial mortality and disability. In 2019, dietary risk factors were responsible for 7.9 million deaths and 187.7 million disability-adjusted life years (DALYs) globally [1]. A poor diet is strongly associated with major diseases such as cardiovascular disease, which is the leading cause of diet-related deaths and disability worldwide. This is followed by cancer and type 2 diabetes, which also significantly contribute to deaths and years of healthy life lost globally. These diseases are largely influenced by dietary factors including high sodium intake and low consumption of whole grains and fruits, highlighting the crucial role of balanced nutrition in preventing these conditions [2].

The World Health Organization (WHO) promotes a diet rich in plant foods such as fruit, vegetables, whole grains, pulses, seeds, and nuts [3]. The EAT-Lancet Commission similarly recommends limiting red and processed meat, added sugar, and highly processed foods, while also stressing that healthy diets must be made accessible through supportive policy measures [4]. Many countries have their own dietary guidelines; in Germany, these are issued by the German Nutrition Society (DGE). Dietary advice should also be culturally sensitive, as eating habits are shaped by tradition, religion, and social norms [5]. Advancing global health will require expanded efforts to embed these dietary and lifestyle practices into everyday life, ensuring that healthy eating becomes accessible, attainable, and sustainable for communities worldwide [6]. Epidemiological evidence and findings from clinical trials consistently indicate that such dietary patterns lower the risk of non-communicable diseases (NCDs), including cardiovascular disease and cancer [7–9].

In light of the growing prevalence of diet-related diseases and their impact on overall health, it is imperative that future medical professionals are equipped with the necessary skills and competencies in this area [10]. Physicians are often the primary point of contact for nutrition and lifestyle matters, they often lack the necessary knowledge and counselling skills to provide practical, informed advice. Patients also express a need for proper advice from their physicians [11], stating that they would adopt a more plant-based diet if it were recommended to them [12]. In order to enable healthcare professionals to promote preventive health effectively and provide holistic, patient-centred care, it is essential to strengthen nutrition education [13]. Four major clinical nutrition societies have declared nutritional care to be a human right [14]. Despite the barriers, physicians themselves view nutrition counselling as important. In order to enhance its role in disease prevention and treatment, there is a need for improved nutrition education, increased funding, and more time allocated to counselling [11].

### Planetary health and nutrition

In addition to the critical role of the microbiome system in health, which is influenced by dietary factors [15], it is becoming increasingly recognised that healthy eating habits and lifestyle choices are essential for promoting the health of individuals and the planet. Sustainable diets, characterised by a predominantly plant-based foods intake, minimal processing and consideration of environmental factors, support human physiological well-being and the preservation of ecological systems [16]. Therefore, given the significant impact that dietary choices have on both personal health outcomes and environmental sustainability, it is essential to have comprehensive nutritional knowledge [17, 18].

### Undergraduate medical education in nutrition

Although the critical importance of nutrition is widely acknowledged, it is still not sufficiently included in medical curricula [19, 20] and prospective medical professionals are not equipped with the necessary skills and competencies in this area [10]. Furthermore, there is a shortage of qualified doctors available to train students in CM [21]. However, several recent studies suggest that CM and TK-teaching are most effective when taught by an interdisciplinary team. Undergraduate students also value this approach [22, 23]. The transdisciplinary nature of the intervention, combined with structured reflection, offers a perspective on the acquisition of skills that can provide students with valuable insights into their ongoing learning [24]. Eisenberg et al. identified 36 essential nutrition competencies for medical education. These cover areas such as knowledge, assessment, communication, public health, collaboration, and referral. In order to close training gaps and improve the provision of holistic care, the authors recommend integrating these competencies into curricula and licensing exams. These recommendations are based on U.S. House Resolution 1118, which calls for nutrition education in medical training in order to combat rising diet-related diseases and healthcare costs. The model could also be adopted internationally [25].

In 2014, only 68.8% of responding European medical schools included mandatory nutrition education, averaging 23.7 h across the curriculum — significantly less than the recommended 25 to 44 h in the US. These findings, alongside the variations between institutions, emphasise the need for an EU-wide curriculum and benchmarks to better prepare doctors to provide evidence-based nutritional care [26].

Overall, nutrition education for German physicians undergoing further training relies heavily on specialist postgraduate programmes, optional courses and interdisciplinary collaborations, rather than being systematically integrated into all continuing medical education programmes. While this approach is gradually improving, it still contrasts with the more structured, competency-based, integrated nutrition education models seen globally [12, 27, 28].

### Experience-based learning as a promising approach to nutrition education

Kolb's (1984) learning cycle illustrates the importance of experience in the learning process. He describes a four-stage model of how knowledge is generated through the cognitive processing of experiences:

1. concrete experience (doing/feeling).
2. reflective observation (observing/reviewing).
3. abstract conceptualisation (thinking/reasoning); and.
4. active experimentation (planning/trying out). The iterative cycle allows learners to potentially enter at any stage and supports four learning styles based on preferences for these modes [29].

Dornan applies this approach to medical education, describing experiential learning as learning that arises from participation in real work or practice, such as patient care. Reflection on these experiences can lead to the development of competence and professional identity. In short, experiential learning is not just about completing tasks, it´s about learning through experience combined with guided reflection [30].

### Nutritional medicine, culinary medicine and teaching kitchen

**Fig. 1:**
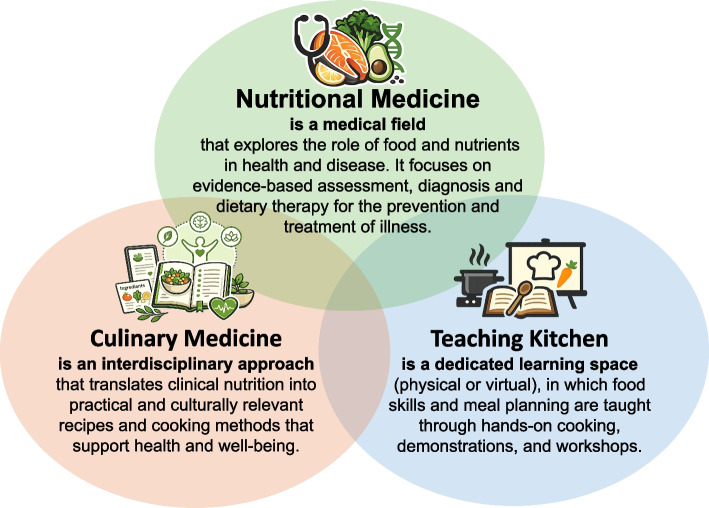
Definition of the three most common courses offered in medical education in Germany. (Authors’ own illustration, created with the support of ChatGPT (OpenAI)

Various approaches have been developed to address gaps in education and qualifications in nutritional medicine. Nutritional medicine (NM) is a specialised field that uses diet- and nutrient-based approaches to promote health, prevent disease, and treat medical conditions. Rather than simply alleviating symptoms, it addresses the underlying nutritional and metabolic disorders [31]. One particularly promising approach is culinary medicine (CM). CM is a form of food-based nutritional education for medical trainees, healthcare professionals, and community members. Classes are conducted using an interactive learning approach, such as problem-based learning. This may or may not involve hands-on activities, such as cooking together. CM's educational content uses food as a practical therapeutic tool to help people choose and enjoy high-quality meals that support disease prevention and treatment and restore wellbeing [32, 33]. The term 'teaching kitchen' (TK) refers to the physical or virtual spaces in which CM is practised. These diverse spaces can be industrial, community, university or home kitchens, and can be used for on-site or synchronous online cooking applications [23] (see Fig. 1). In addition, TK's curriculum integrates topics such as health promotion, counselling communication skills, and the practical application of knowledge in everyday kitchen settings, all within an experience-based learning approach. Particular emphasis is placed on integrating recommendations into a healthy lifestyle and considering psychosocial factors. Learning strategies to support behavioural change are also covered, such as motivational interviewing. TKs offer excellent opportunities to educate professionals and the wider community in nutrition and lifestyle medicine. Meanwhile, they form part of the medical education programme at some universities [34, 35]. Several studies have already demonstrated the positive effects of CM courses on medical students' counselling competencies, nutritional knowledge, and eating habits [36–38]. In addition to these benefits, the latest studies also show that students are highly interested in these courses and that there is high demand for them [10, 33]. While NM is now part of the curriculum at many US faculties, either as CM or TK, there is no evidence regarding its incorporation into German medical curricula. This review aims to investigate this in Germany.

### Objectives

1. How are CM and TK courses implemented at German medical schools, and how widespread are they?
2. How do CM and TK courses improve medical students' acquisition of diet-related competencies?
3. How do students generally receive and evaluate the courses?

## Methods

The authors (BSS, LS, AH) conducted a systematic review in accordance with the Preferred Reporting Items for Systematic Reviews and Meta-Analyses (PRISMA) guideline (version 2020). The PRISMA checklist for abstracts and the full guideline is available in the supplementary material (Tables S1 and S2).

A systematic literature search was conducted between 24 and 30 October 2023 using the search strings and search strategy listed in Table S3. Between 23 July and 30 August 2025, follow-up research in the form of a manual search of reference lists and a search for new publications by known working groups and websites was initiated. The review protocol was registered with PROSPERO: CRD420251109133.

### Data sources and search strategy

The following databases were searched in 2023: CINAHL, Embase, Medline, PubMed, Scopus, LIVIVO, Web of Science, the Cochrane Library, the Deutsche Nationalbibliothek catalogue, Google Scholar and Open Access Theses and Dissertations. Search strings were created in both English and German, since a thorough search of German databases was also conducted. Other methods of identifying studies included contacting authors or experts, checking reference lists, and searching for eligible studies included in the review. No restrictions were placed on the search language or data. All databases, search strings, and results from this first review period are provided in the supplementary material, Table S3.

### Eligibility criteria

This review encompasses randomised and non-randomised interventional studies conducted as part of undergraduate medical education in Germany. Included studies must involve medical students receiving training in NM, CM or TK through formats such as courses, seminars, modules or structured programmes. Studies were included that evaluated interactive or experience-based learning formats.

### Exclusion criteria

Studies evaluating formats consisting solely of lectures were excluded. Studies that did not report empirical data such as questionnaires, open-ended questions or interviews were excluded. No other exclusion criteria were applied with regard to survey instruments, study type, or language.

### Screening and selection process

The findings were imported into Rayyan (Version 1.3.3). After duplicates were deleted, two authors (BSS and LS) independently screened and selected the studies in two stages. In the first stage, the titles, abstracts, and keywords of the articles were screened according to the eligibility criteria provided. Relevant articles progressed to the second stage. Here, the two authors screened the full texts to determine final inclusion or exclusion in the review. Each screening step was conducted in a blinded manner, and any discrepancies and/or conflicts between reviewers were resolved through discussion.

### Data extraction process

One researcher carried out the data extraction (BSS). A second researcher (AH) then performed quality control on the extracted data for each study. The following information about each study was entered into a Microsoft Excel spreadsheet:

1. Basic information:
  Study ID: title, author and year of publication
  Study site(s): where the study was conducted
  Study design
  Intervention: course title, participants, curricular integration (status/semester/teaching kitchen), format and content of the course, teaching methods and number of teaching hours
2. Information on the course evaluation
  Aim(s) of evaluation
  Type of evaluation tools
  Sample size
  Type of data analysis
3. Outcomes of the intervention
  Satisfaction with the teaching unit – the intervention – and estimation of the relevance of the topic by the students
  Data on whether students were able to expand their knowledge of nutrition
  Data on whether students assess that they have developed special skills during the course
  Information on the impact of teaching on attitudes
  Self-assessment by students of behavioural changes or improvements in patient care

### Synthesis methods

As the survey was conducted in the context of medical education, a qualitative and descriptive synthesis of the data was required. Any missing data were disregarded. Heterogeneous statistical outcomes were reported descriptively in terms of the scope, content, delivery and implementation of teaching. No subgroup analyses were performed, nor were any comparisons made between the implementation of individual content and the outcomes. No sensitivity analysis was conducted.

### Study quality assessment

Two researchers (AH and BSS) independently assessed the quality of the included studies independently using the Modified Medical Education Research Study Quality Instrument (MMERSQI) [39]. Although the MMERSQI has a score range of 23.5 to 100, where a higher score indicates better quality, there are currently no guidelines on how to interpret individual scores. It is therefore recommended that comments are made on the various domains to highlight the respective strengths and weaknesses of the articles. The tool covers the following domains: study design; sampling; setting; type of data; validity of the evaluation instrument; data analysis; and outcomes. Any differences in the points awarded by the two researchers were resolved through discussion. Finally, a score was determined for each study and the median and interquartile range (IQR) were calculated across the studies.

## Results

**Fig. 2:**
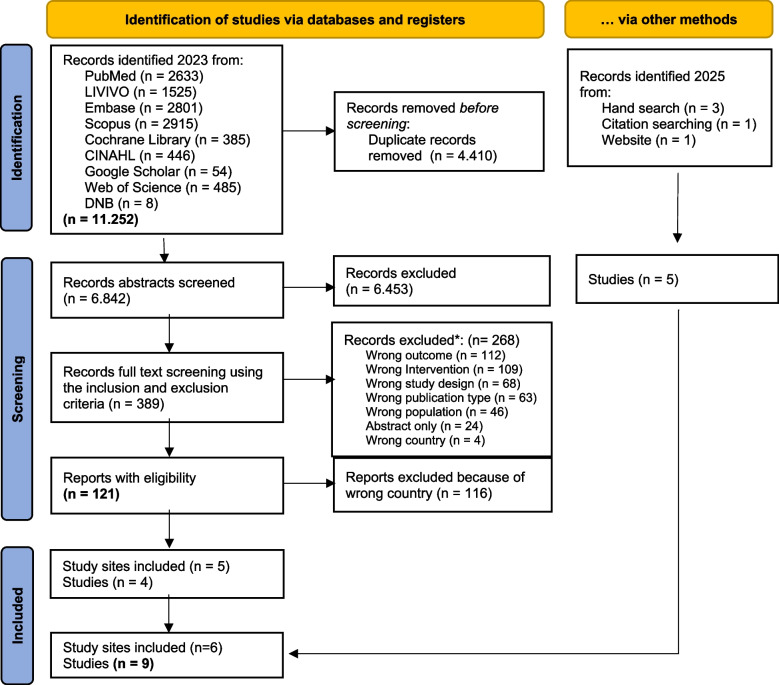
From: Page MJ, McKenzie JE, Bossuyt PM, Boutron I, Hoffmann TC, Mulrow CD, et al. The PRISMA 2020 statement: an updated guideline for reporting systematic reviews. BMJ 2021;372:n71. https://doi.org/10.1136/bmj.n71. *Studies sometimes had several reasons for exclusion

The initial systematic search, conducted between October and December 2023, identified 11,252 records. The systematic process of including and excluding studies according to predefined criteria reduced this number to four. A follow-up search in 2025 identified further five studies: One reported on a new study in Berlin, two were dissertations associated with existing research groups, and two were publications linked to ongoing programmes in Göttingen, Cologne, and Düsseldorf. Ultimately, nine studies met all the inclusion criteria and were included in the final analysis. The study selection process is summarised in Fig. 2 (PRISMA flow diagram).

Following the data extraction scheme described earlier, LS extracted the five studies identified in the initial search, which were then validated by BSS. Subsequent studies were extracted by AH and BSS. Any discrepancies were resolved through discussion.

To answer the first research question, each study was analysed with particular attention to the type of intervention and its characteristics, as well as its integration into the curriculum (Table 1). To answer the second and third questions, the study outcomes and satisfaction levels were categorised and evaluated for significance (Table 2). Finally, the quality of each study was assessed using the MMERSQI (Modified Medical Education Research Study Quality Instrument) (Table 3).

**Table 1:** Study and intervention characteristics

Author/ year	Study site	Study design	Intervention							
			**Participants (n)**	**Title**	**Status/Semester**	**Format**	**Teaching kitchen**	**Course content(s)**	**Teaching methods**	**Number of hours**
Wesselborg, 2019 [40]	Düsseldorf (Medical school HHU + FH)	Cross-sectional	Medical students (25) Nursing students (31)	Interprofessional nutrition management in inpatient and outpatient care – Stop Malnutrition! *	Elective/Semester 5–8	Face-to-face	no	Malnutrition	Case- and research-oriented learning within interprofessional small groups	22.5
Braun, 2019 [41]	Düsseldorf (Medical School HHU + FH)	Longitudinal and prospective cohort intervention	Medical students (22) Nursing students (31)							
Dumm, 2022 [28]	Köln (Medical school)	Controlled cross-sectional and longitudinal	Medical students (325)	Eat This!	Elective/semester 6 onwards	Online synchronous	no	Nutritional medicine, Public health nutrition	Interactive lectures including practical examples for patient counselling and personal diet reflection	10.5
Dumm, 2023 [44]	42 German-speaking medical schools in EU countries	Controlled cross-sectional and longitudinal	Medical students (1531)		Elective/ all semesters	Online synchronous	no	Nutritional medicine, Public health nutrition	Case-based and interactive lectures	22
Helbach, 2023 [45]	39 medical schools in Germany	Controlled cross-sectional and longitudinal	Medical students (1408)							
Böttcher, 2023 [46]	Göttingen (UMG), Brandenburg (MHB), Gießen (JLU)	Longitudinal	Medical students (240)	Culinary Medicine	Elective/clinical study period	Face-to-face (online during the COVID-19 pandemic)	On-site TK, synchronous onloline cooking (during the COVID-19 pandemic)	Nutrition for several indications, malnutrition	Interactive lectures including discussions	21
Rosenau, 2024 [47]	Göttingen, University	Longitudinal	Students from various disciplines, semesters, and degree programs (26)	Planetary Health Diet – Teaching Kitchen Class for a sustainable and healthy Diet	PHD-curriculum	Face-to-face	On-site TK	Planetary health diet	Seminar including students' presentation	21
Drösch, 2025 [48]	Göttingen, medical school	Longitudinal	Medical students (12)	Culinary Medicine	Elective/Semester 5–10	Face-to-face	On-site TK	Healthy diet for the general population, pathologies and special disease patterns	Theoretical part, discussion, cooking and dining	21
Ngoumou, 2025 [49]	Berlin (Charité)	Longitudinal, mixed methods	Medical students (33)	Nutrition and Fasting	Elective/semester 8	Face-to-face	On-site TK	Nutrition and fasting in the context of planetary health	Student-centered, exploratory, content-adaptive teaching, including cooking lessons	37.5

*TK* Teaching kitchen, *HHU* Heinrich Heine University, *FH* Fachhochschule (University of applied sciences), *EU* European Union, *UMG* University Medicine Göttingen, *MHB* Brandenburg Medical School Theodor Fontane, *JLU* Justus-Liebig University; * Studies refer to different focal points for the same intervention

**Table 2:** Intervention evaluation and outcomes

Authors	Aim(s) of evaluation	Evaluation tools	Sample/-size	Data analysis	Outcome				
					**Satisfaction/Course Feedback**	**Knowledge**	**Skills**	**Attitude**	**Behavioural change/Impact on patient care**
Wesselborg, 2019 [40]	Feasibility of interprofessional teaching on malnutrition	Self-developed, validated questionnaire (reliability, factor analysis)	Medical students (25) Nursing students (31)	Inferential statistics (group comparison)	Relevance + + Social climate + + Course Balance + +	n.a	**Application of research-based learning + + + **	n.a	n.a
Braun, 2019 [41]	Impact on A) Interprofessional collaboration B) Malnutrition C) Nutritional management of in-patients	Validated questionnaires: A) Interprofessional collaboration scale, UWE-IP^a^ B) Mini Nutritional Assessment (MNA)^b^ und Grazer Malnutrition Screening (GMS)^c, d^ C) Document analysis: Patient's medical records, doctor's instructions, ward round protocol, nursing documentation and commentary sheets, Complex Care Measures Score, form for taking patient history	A) Students: 28 pre 21 post B) Patients: 169 pre 165 post C) Cases: 104	Inferential statistics	n.a	n.a	n.a	A) Interprofessional collaboration + +	B) Prevalence reduction of malnutrition + C) **Nutritional management** + + +
Dumm, 2022 [28]	Demand for and acceptance of NM teaching	Self-developed questionnaire	Students: 208 pre 113 post 57 CG	Inferential statistics (pre-post comparison)	**Need for NM in general** + + + NM-importance as a competency field in the curriculum + + **NM-importance as a specialist area** + + + **NM-importance as a professorship** + + +	**Learning progress in NM-competencies (self-perceived)** + + +	n.a	**Learning progress regarding preparedness** + + +	n.a
Dumm, 2023 [44]	A) Status quo nutrition B) Effectiveness of teaching	Self-developed + 5 questions adapted from validated questionnaires (Literature reference^e^, reliability, factor analysis)	Students: 1224 pre/ post 122 CG	Inferential statistics (pre—post comparison)	n.a	**NM-competence (self-perceived)** + + + **NM-knowledge (objective)** + + +	**Competence in NM-counselling** + + + Competence in prevention and treatment counselling (-/+)	**Recognising the importance of NM** + + + **Climate crisis awareness** + + +	n.a
Helbach, 2023 [45]	Status quo nutrition and effectiveness of CM teaching on A) Nutrition B) Lifestyle C) Planetary Health	Questionnaires: A) Self-developed, including Items of the short-form Food Frequency Questionnaire (FFQ)^f^ B) Healthy Lifestyle Index (HLI)^g^ C) Self-developed	Students: 520 pre/ post 64 CG	Inferential statistics (pre-post comparison), Multivariate analyses	n.a	n.a	n.a	C) **Healthy Food Awareness** + + + C) **Personal responsibility** + + +	A) **Dietary quality** + + + A) Guideline adherence -/+ B) **Low-Risk lifestyle habits** + + +
Böttcher, 2023 [46]	Effectiveness of online and face-to-face teaching on A) Nutrition B) Counselling competencies C) Well-Being	Questionnaires: A) Self-developed B) Self-developed, literature-based^h^ C) WHO-5-Well-Being Index^i^	Students: 70 virtual 80 in-person	Inferential statistics (pre-post, and teaching format comparison)	n.a	A) Nutrition knowledge (objective) + + **Nutrition knowledge (Comparison in virtual and in person)** + + +	B) **Counselling competencies in virtual and in person** + + +	B) **Attitude towards nutrition counselling** in **virtual and in person** + + + C) **Well-Being in virtual and in person** + + +	B) **Eating habits differ** in some points in person and virtual and **pre and post** + + + A) Cooking frequency -/+
Rosenau, 2024 [47]	Feasibility, effects on the planetary health diet literacy	Questionnaires: A) Official teaching evaluation (ZESS) B) Self-developed, literature-based^h^	Students: 25	A) Descriptive B) Inferential statistics (pre-post comparison)	A) Overall assessment of the course + +	A) Learning gain (self-perceived) + + B) **Health diet literacy (self-perceived)** + + +	n.a	n.a	n.a
Drösch, 2025 [48]	A) Impact on students’ cooking and dietary habits, attitudes, knowledge B) Counselling competencies C) Well-being	Questionnaires: A) Self-developed B) Self-developed literature-based^h^ C) WHO-5-Well-Being Index^i^	Students: 12	Inferential statistics (pre-post comparison)	n.a	A) **Knowledge nutrition therapy (objective)** + + +	B) **Counselling competencies** + + +	A) Relevance of nutrition in medicine -/+ C) Well-being/+	A) Cooking frequency -/+ B) Personal cooking habits -/+ B) Personal dietary habits -/+
Ngoumou, 2025 [49]	Presentation of the development, implementation, and evaluation process	A) Self-developed questionnaires B) Guided interviews	Students: A) 27 B) 8	A) Inferential statistics (pre-post comparison) B) Qualitative	A) Overall rating of the course + + A) Relevance + + A) Experience with module-integrated diet change + + B) Need of evidence-based nutrition knowledge + +	n.a	n.a	A) Effectiveness of nutrition for health promotion, prevention, and treatment + +	n.a

*CG* Control group, *NM* Nutrition Medicine, *ZESS* Central Institution for Language and Key Qualifications of the University of Gottingen, *n.a*. not applicable, ± = no difference measured, + = small difference, + + = large difference, + + + and in bold = significant difference (*p* ≤ 0.05)

^a^Pollard K. UWE-IP University of the West of England Interprofessional Questionnaire. Übersetzung durch die Abteilung Allgemeinmedizin und Versorgungsforschung des Universitätsklinikums Heidelberg. Heidelberg: Universität Heidelberg;

^b^Nestlé Nutrition Institute. Nestlé Nutrition Institute—MNA® Elderly- Overview. 2017. Zugänglich unter/available from: http://www.mna-elderly.com;

^c^Arbeitsgemeinschaft Klinische Ernährung. Ernährungsscores -Screeningbögen. Zugänglich unter/available from: http://www.ake-nutrition.at/SCREENING-BOEGEN.14.0.html;

^d^Roller RE, Eglseer D, Eisenberger A, Wirnsberger GH. The Graz Malnutrition Screening (GMS): a new hospital screening tool for malnutrition. Br J Nutr. 2016;115(4):650–657.; DOI: 10.1017/S0007114515004924

^e^McGaghie WC, Van Horn L, Fitzgibbon M, Telser A, Thompson JA, Kushner RF, et al. Development of a measure of attitude toward nutrition in patient care. Am J Prev Med 2001;20(1):15e20. https://doi.org/10.1016/S0749-3797(00)00264-6

^f^Cleghorn, C.L.; Harrison, R.A.; Ransley, J.K.;Wilkinson, S.; Thomas, J.; Cade, J.E. Can a Dietary Quality Score Derived from a Short-Form FFQ Assess Dietary Quality in UK Adult Population Surveys? Public Health Nutr. 2016, 19, 2915–2923. [CrossRef]

^g^Li, Y.; Pan, A.; Wang, D.D.; Liu, X.; Stampfer, M.; Willett, W.C. The Impact of Healthy Lifestyle Factors on Life Expectancies in the US Population. Circulation 2019, 138, 345–355. [CrossRef]

^h^Razavi, A.C.; Monlezun, D.J.; Sapin, A.; Stauber, Z.; Schradle, K.; Schlag, E.; Dyer, A.; Gagen, B.; McCormack, I.G.; Akhiwu, O.; et al. Multisite Culinary Medicine Curriculum Is Associated with Cardioprotective Dietary Patterns and Lifestyle Medicine

Competencies Among Medical Trainees. Am. J. Lifestyle Med. 2020, 14, 225–233. [CrossRef]

^i^Topp, C.W.; Ostergaard, S.D.; Sondergaard, S.; Bech, P. The WHO-5 Well-Being Index: A systematic review of the literature. Psychother Psychosom 2015, 84, 167–176. [CrossRef] [PubMed]

**Table 3:** Study quality assessment: MMERSQI scores

Study	Study design	Sampling	Setting	Type of data	Validity of evaluation instrument	Data analysis	Outcomes	**Total**
Max. Score	23	10	8	11	15	17	16	100
Wesselborg, 2019 [40]	10	4	5	4	10	13	7	53
Braun, 2019 [41]	9	4	5	11	10	13	16	68
Dumm, 2022 [28]	9	4	5	4	0	13	7	42
Dumm, 2023 [44]	10	0,5	5	11	15	13	16	70,5
Helbach, 2023 [45]	10	0.5	5	11	5	13	16	60
Böttcher,2023 [46]	10	2	5	11	10	13	16	67
Rosenau, 2024 [47]	9	2	5	4	5	13	7	45
Drösch, 2025 [48]	9	4	5	10	5	13	16	62
Ngoumou, 2025 [49]	9	4	5	4	0	13	16	51
Median	**9**	**4**	**5**	**10**	**5**	**13**	**16**	60
IQR	**1**	**2**	**0**	**7**	**5**	**0**	**9**	16

*MMERSQI* Modified Medical Education Research Study Quality Instrument (Al Asmri, M., Haque, M. S., & Parle, J. (2023). A Modified Medical Education Research Study Quality Instrument (MMERSQI) developed by Delphi consensus. *BMC Medical Education*, *23*(1), 63. https://doi.org/10.1186/s12909-023-04033-6); *IQR* Interquartilrange

### Implementation of CM and TK courses at German medical schools

This review included nine studies that were conducted or developed at six different sites. Two of these studies were conducted at the Medical School in Düsseldorf [40, 41], in collaboration with the Fliedner University of Applied Sciences. Medical and nursing students participated in a six-day course designed to teach them how to develop treatment plans for malnourished patients, both in and out of hospital. Based on a research-oriented learning method [42], the didactic concept involved students working in small, multi-professional teams during the initial sessions to define malnutrition, discuss its causes and treatments, and practise using assessment tools and case studies. The students were then introduced to clinical guidelines, expert standards and enteral nutrition strategies. During their field placements, the teams assessed patients' nutritional status and collaborated with practice partners to design evidence-based treatment plans. Interactive learning took place on the ward in an interprofessional setting, without the involvement of a TK.

Three publications examining the impact of an interactive, digital educational format called 'Eat This' were conducted in the Faculty of Medicine in Cologne. The first publication [28] describes the project´s pilot phase, which comprised seven interactive online teaching sessions totalling 10.5 h for 325 medical students. The lectures covered the fundamentals of NM, its clinical applications, and public health nutrition, based on evidence. Interactive elements such as practical examples for patient counselling and personal dietary reflection were used to promote knowledge transfer. The curriculum is based on the 'Guidelines for Nutrition Therapy in Clinic and Practice' (LEKuP) [43], combined with the recommendations from the EAT-Lancet Commission [4, 16]. Positive feedback from participants and the opportunity for all medical faculties to participate in the digital teaching format led to an implementation study [44, 45]. A series of eleven live online lectures, totalling 22 teaching hours, were attended by over 1,500 students from 42 German-speaking medical schools. Designed to be interactive, with practical examples of patient counselling and personal dietary reflection, the lectures provide a comprehensive introduction to CM and can be transferred to all medical schools as they require no additional equipment, such as an onsite TK.

A third working group at the Faculty of Medicine in Göttingen published three studies. They developed a curriculum incorporating CM and TK, in collaboration with medical schools in Brandenburg and Gießen. The first study [46] evaluated the course using a longitudinal study design, incorporating face-to-face and online lessons format from all three study sites, with the participation of 240 medical students. Cooking classes were offered on-site in TK format, or as synchronous online cooking classes during the online teaching phase, with participants joining from their own homes. The course was adapted to focus on the planetary health diet, including student presentations, and a TK and was offered to students from various disciplines at Göttingen [47]. The CM course for medical students in Göttingen was evaluated as part of a doctoral thesis [48]. All studies were designed longitudinally and based on the same curriculum which was adapted according to the course offered and the students participating. The CM curriculum, which is based on LEKuP [43], comprises seven modules focusing on healthy diets, malnutrition, and dietary therapies for various diseases. Each module consists of an interactive lecture and discussion, followed by a hands-on cookery session involving the preparation of five to seven dishes. This is then followed by a tasting session and communal meal, accompanied by a group discussion. The educational team comprises dietitians, trained chefs, student tutors, and a physician. They provide practical, evidence-based nutrition education.

The study at the Charité in Berlin [49] introduced an elective curriculum based on the ‘Six-Step Approach of Kern’ [50]. This two-week programme is taken at the end of the eighth semester of medical education. The course content was developed by a multidisciplinary team, including students, based on a literature review. The programme aims to enhance scientific understanding of nutrition in relation to human and planetary health by offering practical experience of fasting. The evaluation study was longitudinal and used a self-developed questionnaire, as well as interviews with participants. All teaching formats were offered as electives for medical students, mainly during the clinical phase of their studies. The curricula are based on various nutritional fundamentals and have been adapted to the specific circumstances of each study location. They have been developed as a team. In Berlin the most comprehensive teaching is provided, with 37.5 lessons [49], whereas the other programmes were compiled with about 21–22.5 lessons.

Table 1 provides an overview of the main characteristics of all included studies.

### Culinary medicine courses and medical students’ acquisition of diet-related competencies

At the study site in Düsseldorf, which focuses on interprofessional education (IPE) and practical learning with patients, it was revealed that participants greatly appreciated the opportunity to work on real patient cases, which raised their awareness of nutrition. Patient care also benefited from the training, with routine malnutrition screenings becoming standard practice. This enabled at-risk patients to be identified earlier and to receive timely nutritional support. Better documentation of food intake allowed staff to recognise poor eating habits sooner and intervene, thereby reducing the risk of malnutrition-related complications. The investigation yielded significantly more positive results with regard to the skill of applying research-based learning, a skill that nursing students rated higher than medical students [40, 41].

In the Cologne pilot study, participants reported significant improvements in their self-assessed willingness and competence in nutritional counselling and nutrition-related topics, both when compared the pre- and post-group results and when compared to the control group [28]. Further investigation showed that their objective knowledge scores, as measured by 33-item multiple-choice tests, had increased significantly when pre- and post-test scores were compared (p < 0.05). Similarly, self-perceived competence in counselling patients on nutrition-related topics increased, as did positive views on the importance of nutrition [44]. A significant increase in awareness of healthy eating and a healthy lifestyle was also demonstrated. After completing the online lecture series 'Eat This!', awareness of healthy eating increased significantly in the intervention group and adherence to the presented fruit and vegetable guidelines in the students’ own diets rose significantly. More participants also achieved a 'low-risk lifestyle' profile [45].

Studies conducted in Göttingen,Brandenburg and Gießen, demonstrated that the CM elective significantly improved the counselling skills, nutritional knowledge, attitudes towards nutritional counselling, subjective well-being and healthy eating habits of medical students in both virtual and in-person settings. The only parameter that did not change in the before-and-after comparison of the two groups was how often they cooked [46]. Significant improvements in Planetary Health Diet (PHD) competence demonstrated that the PHD curriculum effectively improved participants’ competence in this area, regardless of their field of study [47]. Another study showed significant improvements in nutritional knowledge and counselling skills, as well as moderate improvements in personal cooking skills, dietary behaviour, subjective well-being, and the importance placed on nutrition in medicine [48].

The study from Berlin showed a large difference in the pre post comparison in the effectiveness of nutrition for health promotion, prevention and treatment assessed by the participants [49].

Furthermore, only two studies conducted an objective evaluation: one used patient outcomes to assess learning outcomes [41], while the other employed an objective knowledge test [44]. Regardless of whether instruction took place in a classroom, involved a CM or TK intervention or was an online educational programme, such as the 'Eat This' initiative in Cologne, self-assessed knowledge of nutrition topics increased in the before-and-after comparison across all studies in this review.

Table 2 provides an overview of the outcomes of all the included studies.

### Students’ feedback on the courses

In addition to evaluating the impact on knowledge, skills and attitudes, all studies assessed participants' satisfaction with the course and intervention.

In the Düsseldorf study, nursing and medical students rated the relevance of the 'malnutrition' topic highly after the course, as well as the social climate within the interprofessional team and the balance of the course. The nursing students scored higher on all of these items, but the difference was not statistically significant [40].

The pilot study in Cologne not only demonstrated improved knowledge and attitudes, as evidenced by the pre-post comparison. By the end of the course, a significantly higher proportion of participants stated that NM should be taught as a specialist subject with its own professorships, and that there is a high demand for NM in general [28].

The overall assessment of the course at the study site in Göttingen showed that 75% of participants rated the course as 'very good' and 25% gave four out of five points. Due to the small sample size, no differences were calculated between the different disciplines [47].

On the Berlin course, most participants rated the topic as 'very good' or 'good'. There were slightly more 'very good' ratings in Semester 1, but this difference was not significant. The majority of participants who tried the dietary changes found them helpful, with 84.2% reporting a positive experience. Qualitative feedback also indicated support for the expansion of evidence-based nutrition teaching in medical education [49].

### Quality of the studies included in this review

The MMERSQI was used to evaluate the quality of the studies. The median study quality score was 60 out of a possible 100, with an interquartile range (IQR) of 16. The highest scores were achieved in the 'Setting' (5 out of 8) and 'Quality of Data Analysis' (13 out of 17) domains, while the lowest scores were achieved in the 'Study Design' (9 out of 23) and 'Sampling' (4 out of 10) domains. Six studies achieved the maximum score of 16 for 'outcomes' (see Table 3).

## Discussion

This review provides an overview of the interactive teaching of CM for undergraduates at German medical schools. Following extensive research, nine studies from six study sites were identified that had developed and evaluated training programmes.

### Implementation and dissemination of the courses in Germany

This review reveals that CM and TK courses at German medical faculties offer a variety of implementation options. The interactive teaching methods include practical cooking sessions, discussions, and case-based learning. Face-to-face courses are available for all these methods, as are hybrid formats and synchronous online courses. According to this finding there is evidence that didactic, high-quality online teaching, which provides interactive learning modules [51] and blended learning formats [52] or a combination of online and in-person teaching [53] leads to an increase in competencies.

All identified CM and TK courses are relatively recent developments, with the earliest published in 2019 (Düsseldorf). In their international scoping review on CM in medical schools. Tan et al. (2022) observed that research on this subject has only emerged since 2015, and exclusively in North America [10]. This shows that the field is still in its infancy worldwide. In both North America and Germany, the process is being driven from the ground up. Studies in the USA indicate that there is demand for NM to be incorporated into medical curricula [23]. In 2020, more than 50 university representatives came together to publish a statement emphasising the necessity and feasibility of incorporating nutrition medicine into medical curricula [54]. A student-led initiative in Germany has published a 2025 curriculum intended to serve as a basis for implementation at medical faculties [55].

None of the courses evaluated in this review formed part of the compulsory curriculum. They were all offered on a voluntary basis, often with small group sizes, suggesting that structured teaching in CM only reaches a limited number of medical students in Germany. Although related competencies are scattered across the National Competence-Based Catalogue of Learning Objectives for Medicine [56], CM and TK are not established as distinct subject areas in the curriculum. Several international studies have demonstrated the feasibility and effectiveness of incorporating CM, with or without TK, into the core curriculum, thereby improving medical students' knowledge, skills, attitudes and confidence in counselling patients [23, 37, 57].

In addition to integrating nutrition competencies into the core curriculum, U.S. medical and health professions programmes emphasise the importance of incorporating nutrition knowledge and clinical skills at every stage of the curriculum, as well as the suitability of case-based and problem-oriented learning for teaching clinically relevant, evidence-based nutrition goals [58].

### Impact of culinary medicine education on students´diet-related competencies

The impact of learning more about nutrition on students' lifestyle, competencies and dietary habits has been verified [19, 59]. The studies included in this review demonstrate that CM and TK courses in Germany have a positive impact on self-perceived nutrition competencies (e.g. counselling skills in Göttingen, and competence gains in Cologne), dietary behaviours (e.g. increased cooking frequency and improved habits in Göttingen and Brandenburg), and attitudes (e.g. a greater appreciation of nutrition´s importance and a heightened awareness of planetary health in Cologne and Berlin). These results are in line with international studies, showing that confidence of students in overall culinary skill level increase significantly after the teaching [60, 61].

Several studies suggest that teaching CM can improve practical nutrition learning, as well as confidence and counselling skills, more effectively than nutrition instruction based solely on lectures. A randomised controlled trial at Yale University found that the nutritional knowledge of primary care residents improved through a combination of hands-on CM and lecture-based teaching. However, the hands-on group demonstrated greater confidence and behavioural change across more counselling domains [62]. Furthermore, an interprofessional CM course was found to enhance the confidence and CM-knowledge of healthcare professional students after completing the practical element [22].

When the infrastructure or feasibility of in-person locations is limited, virtual or telemedicine programming offers a convenient and well-received alternative method of carrying out interventions using home kitchen tools or any kind of interactive learning [63]. An outcome which was also demonstrated in the “Eat this” Programme in Cologne [28, 44, 45] and in Göttingen [46].

Together, these findings and the report on behavioural frameworks and translational applications by Krenek [63] provide evidence that CM and culinary nutrition are innovative approaches to influencing dietary behaviour change. They suggest that such programmes could extend beyond the transfer of lecture-based knowledge in order to better support applied learning and behavioural change.

As in this review, most studies refer to self-assessment rather than objective measurement parameters. A pre-post comparison, such as exam results or patient ratings, would provide a more reliable indication of competence gain [64]. In addition to the objectively measurable increase in competence, the potential for significant improvement in interprofessional skills within the team through CM and TK would also be of interest [65].

### Student experiences and course appraisals

The students generally gave the courses very positive ratings in terms of satisfaction, relevance of the topic and feasibility. These course appraisals are to be found in many course evaluations of several medical schools in the US which have successfully integrated CM into their curricula, often using TK formats and interprofessional instruction [33, 66]. Our results highlight significant student interest in this topic and its importance. Students emphasise the importance of nutrition-related training for their future careers, as it equips them with the practical skills and confidence needed for patient counselling. CM programmes support this by combining interactive, hands-on learning with practical work in TK and interdisciplinary collaboration [36]. Above all, students want a course in NM that is not only theoretical and patient-focused, but also provides practical guidance on dietary habits as CM courses do [67].

### Study quality

Typical of medical education research is a moderate methodological quality (median MMERSQI score of 60 points), with low scores for study design, sample selection and setting [68, 69]. Different locations have different offerings, which makes multicentre studies difficult, as comparability between the offerings must first be established [10]. Since participation in the programmes in the studies found was voluntary, randomisation of the groups was difficult, as students could not be forced to participate in or prevented from participating in courses for the purposes of the study. Similarly, group sizes are often too small to allow for group comparisons. This explains why no randomised studies were found in our review, which is quite common in medical education research [70]. It is more important, among other things, that decisions regarding methodology are made prior to the intervention and that a comparison group is included. Nevertheless, group comparisons were carried out in four studies. The high scores in data analysis and results demonstrate that the authors can present meaningful results, at least within their own teaching environment.

### Limitations

This review has methodological and interpretative limitations. A single author conducted the initial screening of article titles and abstracts, increasing the likelihood of errors in the exclusion process. Nevertheless, all full texts were reviewed independently and the included studies were verified by two authors. The data extracted for the two summary tables was verified by both reviewers.

In addition to following the strict protocol of a systematic review, a manual search led to further relevant publications. As the number of projects in Germany is limited to just a few locations, we assume that our overview includes all the projects and curricula currently available to students in the field of CM and TK in Germany.

The uniformly positive ratings for all measured outcomes are noteworthy. However, as these were elective modules, there may be a bias here, as it is possible that only students who were already interested in the subject area chose these courses over others. There is evidence to suggest that courses in which students participate more actively lead to higher ratings [71].

## Conclusion and outlook

Overall, these results demonstrate that innovative, interactive teaching methods in NM, CM and TK can significantly increase medical students' interest in, willingness to engage with, and perceived competence in NM. Future research should focus on identifying competencies, knowledge and skills, as well as appropriate assessment mechanisms, to improve transparency regarding current needs and limitations. These findings could inform the development of future syllabuses and curricula, thereby reinforcing the importance of CM in medical education. Therefore, future curricula at German universities should also incorporate CM within the contexts of prevention and interprofessional learning, as has been successfully implemented at some American universities. Longitudinal studies are needed to measure the long-term effects of teaching. Rather than focusing on self-assessed skills, the endpoint of the survey should be the counselling skills that students have actually acquired in their everyday working lives.

## Supplementary Information

Supplementary Material 1: PRISMA checklist for abstracts and the detailed guideline can be found in the supplementary material (Table S1, S2). All databases, search strings and results are provided in the supplementary material (Table S3).

## Data Availability

All data generated or analysed during this systematic review are included in this published article (extracted data tables) and its supplementary information files (search strategy).

## Abbreviations

CM: Culinary Medicine
DALYs: Disability-Adjusted Life Years
IPE: InterProfessional Education
IQR: InterQuartile Range
LEKuP: Leitfaden Ernährungstherapie in Klinik und Praxis
MMERSQI: Modified Medical Education Research Study Quality Instrument
NCDs: Non-Communicable Diseases
NM: Nutritional Medicine
PRISMA: Preferred Reporting Items for Systematic Reviews and Meta-Analyses
TK: Teaching Kitchen
WHO: World Health Organization

## References

[1] Qiao J, Lin X, Wu Y, Huang X, Pan X, Xu J, et al. Global burden of non-communicable diseases attributable to dietary risks in 1990–2019. J Hum Nutr Diet. 2022;35:202–13. 10.1111/jhn.12904. 33834556 10.1111/jhn.12904

[2] GBD 2017 Diet Collaborators. Health effects of dietary risks in 195 countries, 1990-2017: a systematic analysis for the Global Burden of Disease Study 2017. Lancet. 2019;393:1958–72. 10.1016/S0140-6736(19)30041-8. 30954305 10.1016/S0140-6736(19)30041-8 PMC6899507

[3] World Health Organization. Global action plan for the prevention and control of noncommunicable diseases, 2013-2020. Geneva, Switzerland: World Health Organization; 2013.

[4] Rockström J, Thilsted SH, Willett WC, Gordon LJ, Herrero M, Hicks CC, et al. The EAT-Lancet Commission on healthy, sustainable, and just food systems. Lancet. 2025;406:1625–700. 10.1016/S0140-6736(25)01201-2. 41046857 10.1016/S0140-6736(25)01201-2

[5] Cámara M, Giner RM, González-Fandos E, López-García E, Mañes J, Portillo MP, et al. Food-based dietary guidelines around the world: a comparative analysis to update AESAN Scientific Committee dietary recommendations. Nutrients. 2021. 10.3390/nu13093131. 10.3390/nu13093131 PMC8471688 34579007

[6] Cena H, Calder PC. Defining a healthy diet: evidence for the role of contemporary dietary patterns in health and disease. Nutrients. 2020. 10.3390/nu12020334. 10.3390/nu12020334 PMC7071223 32012681

[7] Shang X, Liu J, Zhu Z, Zhang X, Huang Y, Liu S, et al. Healthy dietary patterns and the risk of individual chronic diseases in community-dwelling adults. Nat Commun. 2023;14:6704. 10.1038/s41467-023-42523-9. 37872218 10.1038/s41467-023-42523-9 PMC10593819

[8] Liang S, Mijatovic J, Li A, Koemel N, Nasir R, Toniutti C, et al. Dietary patterns and non-communicable disease biomarkers: a network meta-analysis and nutritional geometry approach. Nutrients. 2022. 10.3390/nu15010076. 10.3390/nu15010076 PMC9824098 36615733

[9] Gu X, Bui LP, Wang F, Wang DD, Springmann M, Willett WC. Global adherence to a healthy and sustainable diet and potential reduction in premature death. Proc Natl Acad Sci U S A. 2024;121:e2319008121. 10.1073/pnas.2319008121. 39621925 10.1073/pnas.2319008121 PMC11648617

[10] Tan J, Atamanchuk L, Rao T, Sato K, Crowley J, Ball L. Exploring culinary medicine as a promising method of nutritional education in medical school: a scoping review. BMC Med Educ. 2022;22:441. 10.1186/s12909-022-03449-w. 35672843 10.1186/s12909-022-03449-w PMC9175378

[11] Mertens HL, Kaifie A. Ernährungsberatung in ärztlichen Praxen verschiedener Fachrichtungen – eine Querschnittsstudie. [Nutrition counseling in medical practices-a cross-sectional study]. Bundesgesundheitsbl Gesundheitsforsch Gesundheitsschutz. 2024;67:721–9. 10.1007/s00103-024-03870-0. 10.1007/s00103-024-03870-0 PMC11166768 38639815

[12] Hanslian E, Schiele JK, Jeitler M, Michalsen A, Wischnewsky M, Storz MA, et al. Attitudes toward healthy nutrition in Germany - results from an online-representative cross-sectional survey. Front Nutr. 2024;11:1480980. 10.3389/fnut.2024.1480980. 39897537 10.3389/fnut.2024.1480980 PMC11783846

[13] Khiri N, Howells K. Nutritional education in medical curricula and clinical practice: a scoping review on the knowledge deficit amongst medical students and doctors. J Hum Nutr Diet. 2025;38:e70031. 10.1111/jhn.70031. 40047058 10.1111/jhn.70031 PMC11883500

[14] Cárdenas D, Toulson Davisson Correia MI, Hardy G, Ochoa JB, Barrocas A, Hankard R, et al. Nutritional care is a human right: Translating principles to clinical practice. Clin Nutr. 2022;41:1613–8. 10.1016/j.clnu.2022.03.021. 35637040 10.1016/j.clnu.2022.03.021

[15] Ross FC, Patangia D, Grimaud G, Lavelle A, Dempsey EM, Ross RP, et al. The interplay between diet and the gut microbiome: implications for health and disease. Nat Rev Microbiol. 2024;22:671–86. 10.1038/s41579-024-01068-4. 39009882 10.1038/s41579-024-01068-4

[16] Willett W, Rockström J, Loken B, Springmann M, Lang T, Vermeulen S, et al. Food in the Anthropocene: the EAT-Lancet Commission on healthy diets from sustainable food systems. Lancet. 2019;393:447–92. 30660336 10.1016/S0140-6736(18)31788-4

[17] Bodirsky BL, Beier F, Humpenöder F, Leip D, Crawford MS, Chen DM-C, et al. A food system transformation pathway reconciles 1.5 °C global warming with improved health, environment and social inclusion. Nat Food. 2025;6:1133–52. 10.1038/s43016-025-01268-y. 41420095 10.1038/s43016-025-01268-y PMC12716996

[18] Springmann M, Wiebe K, Mason-D’Croz D, Sulser TB, Rayner M, Scarborough P. Health and nutritional aspects of sustainable diet strategies and their association with environmental impacts: a global modelling analysis with country-level detail. Lancet Planet Health. 2018;2:e451–61. 10.1016/S2542-5196(18)30206-7. 30318102 10.1016/S2542-5196(18)30206-7 PMC6182055

[19] Crowley J, Ball L, Hiddink GJ. Nutrition in medical education: a systematic review. Lancet Planet Health. 2019;3:e379–89. 10.1016/S2542-5196(19)30171-8. 31538623 10.1016/S2542-5196(19)30171-8

[20] Thircuir S, Chen NN, Madsen KA. Addressing the gap of nutrition in medical education: experiences and expectations of medical students and residents in France and the United States. Nutrients. 2023;15:5054. 10.3390/nu15245054. 38140313 10.3390/nu15245054 PMC10745340

[21] Blunt SB, Kafatos A. Clinical nutrition education of doctors and medical students: solving the Catch 22. Adv Nutr. 2019;10:345–50. 10.1093/advances/nmy082. 30624632 10.1093/advances/nmy082 PMC6416044

[22] Brennan BR, Beals KA, Burns RD, Chow CJ, Locke AB, Petzold MP, et al. Impact of culinary medicine course on confidence and competence in diet and lifestyle counseling, interprofessional communication, and health behaviors and advocacy. Nutrients. 2023;15:4157. 10.3390/nu15194157. 37836442 10.3390/nu15194157 PMC10574678

[23] Thomas OW, Reilly JM, Wood NI, Albin J. Culinary medicine: needs and strategies for incorporating nutrition into medical education in the United States. J Med Educ Curric Dev. 2024;11:23821205241249379. 10.1177/23821205241249379. 38711830 10.1177/23821205241249379 PMC11072074

[24] Radzikowski JL, Houghton N, Chacon CS, Armstrong-Scott O, Youssef J, Spivey AC, et al. Medical kitchen: transdisciplinary clinical skills training. Clin Teach. 2025;22:e70065. 10.1111/tct.70065. 40056028 10.1111/tct.70065 PMC11889704

[25] Eisenberg DM, Cole A, Maile EJ, Salt M, Armstrong E, Broad Leib E, et al. Proposed nutrition competencies for medical students and physician trainees: a consensus statement. JAMA Netw Open. 2024;7:e2435425. 10.1001/jamanetworkopen.2024.35425. 39348126 10.1001/jamanetworkopen.2024.35425

[26] Chung M, van Buul VJ, Wilms E, Nellessen N, Brouns FJPH. Nutrition education in European medical schools: results of an international survey. Eur J Clin Nutr. 2014;68:844–6. 10.1038/ejcn.2014.75. 24781690 10.1038/ejcn.2014.75

[27] Storz MA, Oksche A, Schlasius-Ratter U, Schillings V, Beckschulte K, Huber R. Nutrition coverage in medical licensing examinations in Germany: an analysis of six nationwide exams. Nutrients. 2022;14:5333. 10.3390/nu14245333. 36558492 10.3390/nu14245333 PMC9780865

[28] Dumm M, Helbach A, Schad E, Polidori MC, Matthes J. Nutrition in medical studies:“Eat This!” a digital teaching concept as an elective subject for German curricula. Piloting a course in Cologne. Ernaehrungsumschau. 2022;69(1):2–9.

[29] Kolb DA. Experimental learning: experience as the source of learning and development. Englewood Cliffs, N.J.: Prentice-Hall; 1984.

[30] Dornan T, Conn R, Monaghan H, Kearney G, Gillespie H, Bennett D. Experience based learning (ExBL): clinical teaching for the twenty-first century. Med Teach. 2019;41:1098–105. 10.1080/0142159X.2019.1630730. 31382787 10.1080/0142159X.2019.1630730

[31] Meldrum JM. What is nutritional medicine? Nutr Health. 1993;9:135–50. 10.1177/026010609300900209. 8134026 10.1177/026010609300900209

[32] La Puma J. What is Culinary Medicine and what does it do? Popul Health Manag. 2016;19:1–3. 10.1089/pop.2015.0003. 26035069 10.1089/pop.2015.0003 PMC4739343

[33] Newman C, Yan J, Messiah SE, Albin J. Culinary Medicine as innovative nutrition education for medical students: a scoping review. Acad Med. 2023;98:274–86. 10.1097/ACM.0000000000004895. 35921151 10.1097/ACM.0000000000004895

[34] Eisenberg DM, Pacheco LS, McClure AC, McWhorter JW, Janisch K, Massa J. Perspective: Teaching Kitchens: conceptual origins, applications and potential for impact within Food Is Medicine research. Nutrients. 2023. 10.3390/nu15132859. 10.3390/nu15132859 PMC10343805 37447185

[35] Razavi AC, Latoff A, Dyer A, Albin JL, Artz K, Babcock A, et al. Virtual teaching kitchen classes and cardiovascular disease prevention counselling among medical trainees. BMJ Nutr Prev Health. 2023;6:6–13. 10.1136/bmjnph-2022-000477. 10.1136/bmjnph-2022-000477 PMC10407392 37559965

[36] Wattick RA, Saurborn EG, Olfert MD. Impact of a brief Culinary Medicine elective on medical students’ nutrition knowledge, self-efficacy, and attitudes. Med Sci Educ. 2022;32:785–92. 10.1007/s40670-022-01566-1. 36035541 10.1007/s40670-022-01566-1 PMC9411439

[37] D’Adamo CR, Workman K, Barnabic C, Retener N, Siaton B, Piedrahita G, et al. Culinary Medicine training in core medical school curriculum improved medical student nutrition knowledge and confidence in providing nutrition counseling. Am J Lifestyle Med. 2022;16:740–52. 10.1177/15598276211021749. 36389046 10.1177/15598276211021749 PMC9644147

[38] Krumholz S, Hurd M, Tenney A, Iacono L, Vollbrecht C, Calkins W. Cooking with the curriculum: a pilot culinary medicine program at the Larner College of Medicine. BMC Med Educ. 2025;25:517. 10.1186/s12909-025-07103-z. 40217237 10.1186/s12909-025-07103-z PMC11987183

[39] Al Asmri M, Haque MS, Parle J. A modified medical education research study quality instrument (MMERSQI) developed by Delphi consensus. BMC Med Educ. 2023;23:63. 10.1186/s12909-023-04033-6. 36698117 10.1186/s12909-023-04033-6 PMC9878889

[40] Wesselborg B, Hoenen M, Adam-Paffrath R, Kuske S, Schendel L, Grünewald M, et al. Interprofessional nutrition management - implementation and evaluation of a course for medical and nursing students using research-based learning method. GMS J Med Educ. 2019;36:Doc68. 10.3205/zma001276. 10.3205/zma001276 PMC6905358 31844640

[41] Braun B, Grünewald M, Adam-Paffrath R, Wesselborg B, Wilm S, Schendel L, et al. Impact of interprofessional education for medical and nursing students on the nutritional management of in-patients. GMS J Med Educ. 2019;36:Doc11. 10.3205/zma001219. 10.3205/zma001219 PMC6446465 30993169

[42] Wildt J. Forschendes Lernen: Lernen im „Format “der Forschung. journal hochschuldidaktik. 20.2 (2009):4–7.

[43] Hauner H, Beyer-Reiners E, Bischoff G, Breidenassel C, Ferschke M, Gebhardt A, et al. Leitfaden Ernährungstherapie in Klinik und Praxis (LEKuP). Aktuel Ernahrungsmed. 2019;44:384–419. 10.1055/a-1030-5207.

[44] Dumm M, Moll K, Helbach A, Leineweber CG, Böttrich T, Ruhtenberg CS, et al. Implementing nutritional medicine into medical curricula: a student-initiated course improves knowledge and attitudes. Clin Nutr ESPEN. 2023;57:181–9. 10.1016/j.clnesp.2023.06.043. 37739654 10.1016/j.clnesp.2023.06.043

[45] Helbach A, Dumm M, Moll K, Böttrich T, Leineweber CG, Mueller W, et al. Improvement of dietary habits among German medical students by attending a nationwide online lecture series on nutrition and Planetary Health (“Eat This!”). Nutrients. 2023. 10.3390/nu15030580. 10.3390/nu15030580 PMC9920441 36771284

[46] Böttcher S, Schonebeck LJ, Drösch L, Plogmann AM, Leineweber CG, Puderbach S, et al. Comparison of effectiveness regarding a Culinary Medicine elective for medical students in Germany delivered virtually versus in-person. Nutrients. 2023. 10.3390/nu15194281. 10.3390/nu15194281 PMC10574049 37836565

[47] Rosenau N, Neumann U, Hamblett S, Ellrott T. University students as change agents for health and sustainability: a pilot study on the effects of a teaching kitchen-based Planetary Health Diet curriculum. Nutrients. 2024. 10.3390/nu16040521. 10.3390/nu16040521 PMC10892560 38398844

[48] Drösch L. Culinary Medicine–A new teaching approach to improve medical students' counselling skills for nutrition-associated diseases: Review and exploratory pilot study at Göttingen medical school [dissertation]. Göttingen: University Medical Center Göttingen; 2025.

[49] Ngoumou GB, Koppold DA, Wenzel L, Schirmaier A, Breinlinger C, Pörtner LM, et al. An interactive course program on nutrition for medical students: interdisciplinary development and mixed-methods evaluation. BMC Med Educ. 2025;25:115. 10.1186/s12909-024-06596-4. 39849444 10.1186/s12909-024-06596-4 PMC11761204

[50] Thomas PA. Curriculum development for medical education: A six-step approach. 4th ed. Baltimore: Johns Hopkins University Press; 2022.

[51] Saiyad S, Virk A, Mahajan R, Singh T. Online teaching in medical training: establishing good online teaching practices from cumulative experience. Int J Appl Basic Med Res. 2020;10:149–55. 10.4103/ijabmr.IJABMR_358_20. 33088735 10.4103/ijabmr.IJABMR_358_20 PMC7534709

[52] Regmi A, Mao X, Qi Q, Tang W, Yang K. Students’ perception and self-efficacy in blended learning of medical nutrition course: a mixed-method research. BMC Med Educ. 2024;24:1411. 10.1186/s12909-024-06339-5. 39627743 10.1186/s12909-024-06339-5 PMC11616338

[53] Dreyer T, Papadopoulos S, Wiesner R, Karay Y. Classroom teaching versus online teaching in physiology practical course - does this lead to different examination results? GMS J Med Educ. 2025;42:Doc8. 10.3205/zma001732. 40395964 10.3205/zma001732 PMC12086253

[54] Cuerda C, Muscaritoli M, Krznaric Z, Pirlich M, van Gossum A, Schneider S, et al. Nutrition education in medical schools (NEMS) project: joining ESPEN and university point of view. Clin Nutr. 2021;40:2754–61. 10.1016/j.clnu.2021.03.010. 33933741 10.1016/j.clnu.2021.03.010

[55] Hill A, Leineweber CG, Wechsler J, Stehle P, Ellerts A, Sester L, et al. Ernährungsmedizin im Studium der Humanmedizin –Mustercurriculum. Aktuel Ernahrungsmed. 2025;50:354–63. 10.1055/a-2635-0093.

[56] Medizinischer Fakultätentag (MFT). Nationaler Kompetenzbasierter Lernzielkatalog Medizin (NKLM) Version 2.0. 2021. https://nklm.de/. Accessed 30 Jan 2026.

[57] Johnston EA, Torres M, Goldgraben S, Burns CM. Integrating nutrition and culinary medicine into preclinical medical training. BMC Med Educ. 2024;24:959. 10.1186/s12909-024-05795-3. 39227833 10.1186/s12909-024-05795-3 PMC11373231

[58] Dimaria-Ghalili RA, Edwards M, Friedman G, Jaferi A, Kohlmeier M, Kris-Etherton P, et al. Capacity building in nutrition science: revisiting the curricula for medical professionals. Ann N Y Acad Sci. 2013;1306:21–40. 10.1111/nyas.12334. 24329516 10.1111/nyas.12334

[59] Mogre V, Stevens FCJ, Aryee PA, Amalba A, Scherpbier AJJA. Why nutrition education is inadequate in the medical curriculum: a qualitative study of students’ perspectives on barriers and strategies. BMC Med Educ. 2018;18:26. 10.1186/s12909-018-1130-5. 29433505 10.1186/s12909-018-1130-5 PMC5809975

[60] Jaroudi SS, Sessions WS, Wang VS, Shriver JL, Helekar AS, Santucci M, et al. Impact of culinary medicine elective on medical students’ culinary knowledge and skills. Proc (Bayl Univ Med Cent). 2018;31:439–42. 10.1080/08998280.2018.1473742. 30948975 10.1080/08998280.2018.1473742 PMC6414012

[61] Pang B, Memel Z, Diamant C, Clarke E, Chou S, Gregory H. Culinary medicine and community partnership: hands-on culinary skills training to empower medical students to provide patient-centered nutrition education. Med Educ Online. 2019;24:1630238. 10.1080/10872981.2019.1630238. 31248353 10.1080/10872981.2019.1630238 PMC6609327

[62] Wood NI, Fussell M, Benghiat E, Silver L, Goldstein M, Ralph A, et al. A randomized controlled trial of a culinary medicine intervention in a virtual teaching kitchen for primary care residents. J Gen Intern Med. 2025;40:2668–78. 10.1007/s11606-025-09652-x. 40562884 10.1007/s11606-025-09652-x PMC12405083

[63] Krenek AM, Mobley AR, Andrade J, Dahl W, Mathews AE. Behavioral Frameworks and Translational Applications of Culinary Medicine and Culinary Nutrition. J Nutr Educ Behav. 2024;56:742–50. 10.1016/j.jneb.2024.07.001. 39152977 10.1016/j.jneb.2024.07.001

[64] Gabbard T, Romanelli F. The Accuracy of Health Professions Students’ Self-Assessments Compared to Objective Measures of Competence. Am J Pharm Educ. 2021;85:8405. 10.5688/ajpe8405. 34283796 10.5688/ajpe8405 PMC8086612

[65] van Horn L, Lenders CM, Pratt CA, Beech B, Carney PA, Dietz W, et al. Advancing Nutrition Education, Training, and Research for Medical Students, Residents, Fellows, Attending Physicians, and Other Clinicians: Building Competencies and Interdisciplinary Coordination. Adv Nutr. 2019;10:1181–200. 10.1093/advances/nmz083. 31728505 10.1093/advances/nmz083 PMC6855992

[66] Lawrence JC, Knol LL, Clem J, de La OR, Henson CS, Streiffer RH. Integration of Interprofessional Education (IPE) Core Competencies Into Health Care Education: IPE Meets Culinary Medicine. J Nutr Educ Behav. 2019;51:510–2. 10.1016/j.jneb.2019.01.013. 30824198 10.1016/j.jneb.2019.01.013

[67] Kicklighter JR, Koonce VJ, Rosenbloom CA, Commander NE. College freshmen perceptions of effective and ineffective aspects of nutrition education. J Am Coll Health. 2010;59:98–104. 10.1080/07448481.2010.483709. 20864435 10.1080/07448481.2010.483709

[68] Li Y, Sun C, Wang Y, Chi H, Tang H, Ma M, et al. Bias in student evaluations of teaching in undergraduate medical education: a qualitative study from a medical school in Northern China. BMC Med Educ. 2025;25:784. 10.1186/s12909-025-07300-w. 40426114 10.1186/s12909-025-07300-w PMC12107909

[69] Reed DA, Cook DA, Beckman TJ, Levine RB, Kern DE, Wright SM. Association between funding and quality of published medical education research. JAMA. 2007;298:1002–9. 10.1001/jama.298.9.1002. 17785645 10.1001/jama.298.9.1002

[70] Cook DA, Reed DA. Appraising the quality of medical education research methods: the Medical Education Research Study Quality Instrument and the Newcastle-Ottawa Scale-Education. Acad Med. 2015;90:1067–76. 10.1097/ACM.0000000000000786. 26107881 10.1097/ACM.0000000000000786

[71] Sullivan GM. Getting off the “gold standard”: randomized controlled trials and education research. J Grad Med Educ. 2011;3:285–9. 10.4300/JGME-D-11-00147.1. 22942950 10.4300/JGME-D-11-00147.1 PMC3179209

