# Supplementary material for: How does culinary medicine training impact the diet-related knowledge, skills and attitudes of undergraduate medical students in Germany?—A systematic review
Source: BMC Med Educ. 2026 Jun 3;26:895. doi: 10.1186/s12909-026-09580-2 (PMC13231769; doi:10.1186/s12909-026-09580-2)
Supplement: Supplementary file 1 — Supplementary Material 1: PRISMA checklist for abstracts and the detailed guideline can be found in the supplementary material (Table S1, S2). All databases, search strings and results are provided in the supplementary material (Table S3). [file 12909_2026_9580_MOESM1_ESM.zip › Table S3.docx]

Table S3: All databases, search strings and results used for the review

| **Date/Name** | **Database** | **Searchstring** | **Hits** |
| --- | --- | --- | --- |
| 24.10.2023 | PubMED | (((("*Kitchen") OR ("Cook*") OR ("*nutrition*") OR ("food as medicine") OR ("culinary*") OR ("Cuisine") OR ("diet")) AND (("program") OR ("course") OR ("*education") OR ("training*") OR ("curriculum") OR ("seminar") OR ("teaching") OR ("module"))) AND (("student") OR ("undergraduate"))) AND (medical) | 2633 |
| 25.10.2023 | LIVIVO | (((("*Kitchen") OR ("Cook*") OR ("*nutrition*") OR ("food as medicine") OR ("culinary*") OR ("Cuisine") OR ("diet")) AND (("program") OR ("course") OR ("*education") OR ("training*") OR ("curriculum") OR ("seminar") OR ("teaching") OR ("module"))) AND (("student") OR ("undergraduate"))) AND (medical) | 1166 |
| 03.11.2023 | Embase | (((("*Kitchen") OR ("Cook*") OR ("*nutrition*") OR ("food as medicine") OR ("culinary*") OR ("Cuisine") OR ("diet")) AND (("program") OR ("course") OR ("*education") OR ("training*") OR ("curriculum") OR ("seminar") OR ("teaching") OR ("module"))) AND (("student") OR ("undergraduate"))) AND (medical)  OR  (((("*Kitchen") OR ("Cook*") OR ("*nutrition*") OR ("food as medicine") OR ("culinary*") OR ("Cuisine") OR ("diet")) AND (("program") OR ("course") OR ("*education") OR ("training*") OR ("curriculum") OR ("seminar") OR ("teaching") OR ("module"))) AND (("student") OR ("undergraduate"))) AND (medical) AND (GERMANY OR SWISS OR SWIZERLAND OR AUSTRIA) | 2765  36  = 2801 |
| 24.10.2023 | SCOPUS | (((("*Kitchen") OR ("Cook*") OR ("*nutrition*") OR ("food as medicine") OR ("culinary*") OR ("Cuisine") OR ("diet")) AND (("program") OR ("course") OR ("*education") OR ("training*") OR ("curriculum") OR ("seminar") OR ("teaching") OR ("module"))) AND (("student") OR ("undergraduate"))) AND (medical)  **Search within Article Title, Abstract or Keywords** | 2915 |
| 24.10.2023 | Cochrane Library | (((("*Kitchen") OR ("Cook*") OR ("*nutrition*") OR ("food as medicine") OR ("culinary*") OR ("Cuisine") OR ("diet")) AND (("program") OR ("course") OR ("*education") OR ("training*") OR ("curriculum") OR ("seminar") OR ("teaching") OR ("module"))) AND (("student") OR ("undergraduate"))) AND (medical) | 385 |
| 24.10.2023 | CINAHL | (((("*Kitchen") OR ("Cook*") OR ("*nutrition*") OR ("food as medicine") OR ("culinary*") OR ("Cuisine") OR ("diet")) AND (("program") OR ("course") OR ("*education") OR ("training*") OR ("curriculum") OR ("seminar") OR ("teaching") OR ("module"))) AND (("student") OR ("undergraduate"))) AND (medical) | 446 |
| 26.10.2023 | Google Scholar | (((("*Kitchen") OR ("Cook*") OR ("*nutrition*") OR ("food as medicine") OR ("culinary*") OR ("Cuisine") OR ("diet")) AND (("program") OR ("course") OR ("*education") OR ("training*") OR ("curriculum") OR ("seminar") OR ("teaching") OR ("module"))) AND (("student") OR ("undergraduate"))) AND (medical)  OR  (((("*Kitchen") OR ("Cook*") OR ("*nutrition*") OR ("food as medicine") OR ("culinary*") OR ("Cuisine") OR ("diet")) AND (("program") OR ("course") OR ("*education") OR ("training*") OR ("curriculum") OR ("seminar") OR ("teaching") OR ("module"))) AND (("student") OR ("undergraduate"))) AND (medical) AND (GERMANY OR SWISS OR SWIZERLAND OR AUSTRIA) | 1.630.000  551.000  = 45 |
| 30.10.2023 | Web of Science | (((("*Kitchen") OR ("Cook*") OR ("*nutrition*") OR ("food as medicine") OR ("culinary*") OR ("Cuisine") OR ("diet")) AND (("program") OR ("course") OR ("*education") OR ("training*") OR ("curriculum") OR ("seminar") OR ("teaching") OR ("module"))) AND (("student") OR ("undergraduate"))) AND (medical)  **Search within Article Title, Abstract or Keywords** | 485 |
| 30.10.2023 | Open Access Theses and Dissertations | (((("*Kitchen") OR ("Cook*") OR ("*nutrition*") OR ("food as medicine") OR ("culinary*") OR ("Cuisine") OR ("diet")) AND (("program") OR ("course") OR ("*education") OR ("training*") OR ("curriculum") OR ("seminar") OR ("teaching") OR ("module"))) AND (("student") OR ("undergraduate"))) AND (medical) | 0 |
| **Deutsche Ergebnisse** | | | |
| 26.10.2023 | Google Scholar | ("*Küche" OR "Koch*" OR "*Ernährung*" OR "Essen*" OR "gesundes" OR "Gesundheit*") AND ("Programm" OR "Kurs" OR "*Bildung" OR "Schulung" OR "Curriculum" OR "Lehrplan" OR "Beratung") AND ("Student*" OR "Medizinstudent*" OR "Studierend*") AND ("Medizin*") AND ("Deutschland")  OR  Ernährung Medizinstudierende Vorlesung  **Nach Interesse ausgewählt** | 27.600  1.170  = 9 |
| 25.10.2023 | LIVIVO | (((("*Küche") OR ("Koch*") OR ("*Ernährung*") OR ("Essen*") OR ("gesundes") OR ("Gesundheit*")) AND (("Programm") OR ("Kurs") OR ("*Bildung") OR ("Schulung") OR ("Curriculum") OR ("Lehrplan") OR ("Beratung"))) AND (("Student*") OR ("Medizinstudent*") OR ("Studierend*"))) AND ("Medizin*") AND ("Deutschland")  OR  (((("*Küche") OR ("Koch*") OR ("*Ernährung*") OR ("Essen*") OR ("gesundes") OR ("Gesundheit*")) AND (("Programm") OR ("Kurs") OR ("*Bildung") OR ("Schulung") OR ("Curriculum") OR ("Lehrplan") OR ("Beratung"))) AND (("Student*") OR ("Medizinstudent*") OR ("Studierend*"))) AND ("Medizin*")  OR  (Ernährung AND Küche AND Students AND Medizin) | 19  153  24  = 206 |
| 26.10.2023 | Katalog der deutschen Nationalbibliothek | (((("*Kitchen") OR ("Cook*") OR ("*nutrition*") OR ("food as medicine") OR ("culinary*") OR ("Cuisine") OR ("diet")) AND (("program") OR ("course") OR ("*education") OR ("training*") OR ("curriculum") OR ("seminar") OR ("teaching") OR ("module"))) AND (("student") OR ("undergraduate"))) AND (medical)  OR  ("*Küche" OR "Koch*" OR "*Ernährung*" OR "Essen*" OR "gesundes" OR "Gesundheit*") AND ("Programm" OR "Kurs" OR "*Bildung" OR "Schulung" OR "Curriculum" OR "Lehrplan" OR "Beratung") AND ("Student*" OR "Medizinstudent*" OR "Studierend*") AND ("Medizin*") AND ("Deutschland")  OR  ("*Küche" OR "Koch*" OR "*Ernährung*" OR "Essen*" OR "gesundes" OR "Gesundheit*") AND ("Programm" OR "Kurs" OR "*Bildung" OR "Schulung" OR "Curriculum" OR "Lehrplan" OR "Beratung") AND ("Student*" OR "Medizinstudent*" OR "Studierend*") AND ("Medizin*") AND ("Deutschland") | 5  15  85  = 8 |
